# Malignant Gastrointestinal Neuroectodermal Tumor in the Right Heart: A Report of an Extremely Rare Case Presenting With a Cardiac Mass

**DOI:** 10.3389/fcvm.2021.702215

**Published:** 2021-09-01

**Authors:** Zhiwen Li, Xiaohong Pu, Lu He, Yao Fu, Lin Li, Yuemei Xu, Wenyan Guan, Xiangshan Fan

**Affiliations:** ^1^Department of Pathology, The Affiliated Drum Tower Hospital, Nanjing University Medical School, Nanjing, China; ^2^Department of Pathology, Nanjing First Hospital, Nanjing Medical University, Nanjing, China

**Keywords:** cardiac malignancy, malignant gastrointestinal neuroectodermal tumor, extra-gastrointestinal, EWSR1-ATF1, case report

## Abstract

Malignant gastrointestinal neuroectodermal tumor (GNET) is an extremely rare soft tissue sarcoma and has been designated as a new entity recently. At present, GNET virtually exclusively occurs in the gastrointestinal tract. Here we report a case of extra-GNET that arose in the right heart. A 62-year-old male complained of chest distress and breathing difficulty while lying down at night for over 1 month at admission. The radiological findings revealed an occupying lesion involving the right atrium and the right ventricle without any abdominal abnormalities. The patient then underwent a surgical resection. Microscopically, neoplastic cells proliferated in the pattern of nests and sheets with fibrous separation. Focal areas with cellular dyscohesion imparted a vague pseudopapillary pattern. These tumor cells were small to medium in size with fine chromatin and predominantly pale eosinophilic cytoplasm. The nuclei were typically round to oval with somewhat irregular contours and contained small nucleoli. The mitotic figures were easily found. Immunohistochemically, the neoplastic cells were positive for S100 and SOX-10 but negative for HMB-45, A103, and CD99. *EWSR1*–*AFTF1* rearrangement was detected by fluorescence *in situ* hybridization and further confirmed by whole-transcriptome sequence analysis. The patient had pulmonary metastasis 8 months later and soon died of the disease. The overall survival of the patient was 20 months. In summary, we reported an extremely rare case of cardiac GNET, indicating that the location of GNET should not be confined to the GI tract as initially defined. Due to the lack of a specific effective treatment and the occurrence of early metastasis, cardiac GNET conferred a poor prognosis. More clinical and experimental studies are warranted to better manage this disease in the future.

## Introduction

Malignant gastrointestinal neuroectodermal tumor (GNET), a synonym for clear cell sarcoma (CCS)-like tumor of the gastrointestinal (GI) tract, is a very rare mesenchymal tumor first described by Zambrano et al. (1). It is usually characterized by an aggressive clinical course and a high rate of local recurrence and metastases (2). Primary GNET almost always occurs in the GI tract, with the small intestine, stomach, and colon being most commonly involved (3). So far, extra-GNET has been reported only in several case reports, all of which presented in the upper aerodigestive tract (4). Here we reported a case of GNET originating from the right ventricle and later metastasizing to the pulmonary that shared similar morphological, immunophenotypic, and molecular characteristics with GNET arising in the GI tract.

## Case Presentation

**Figure 1:**
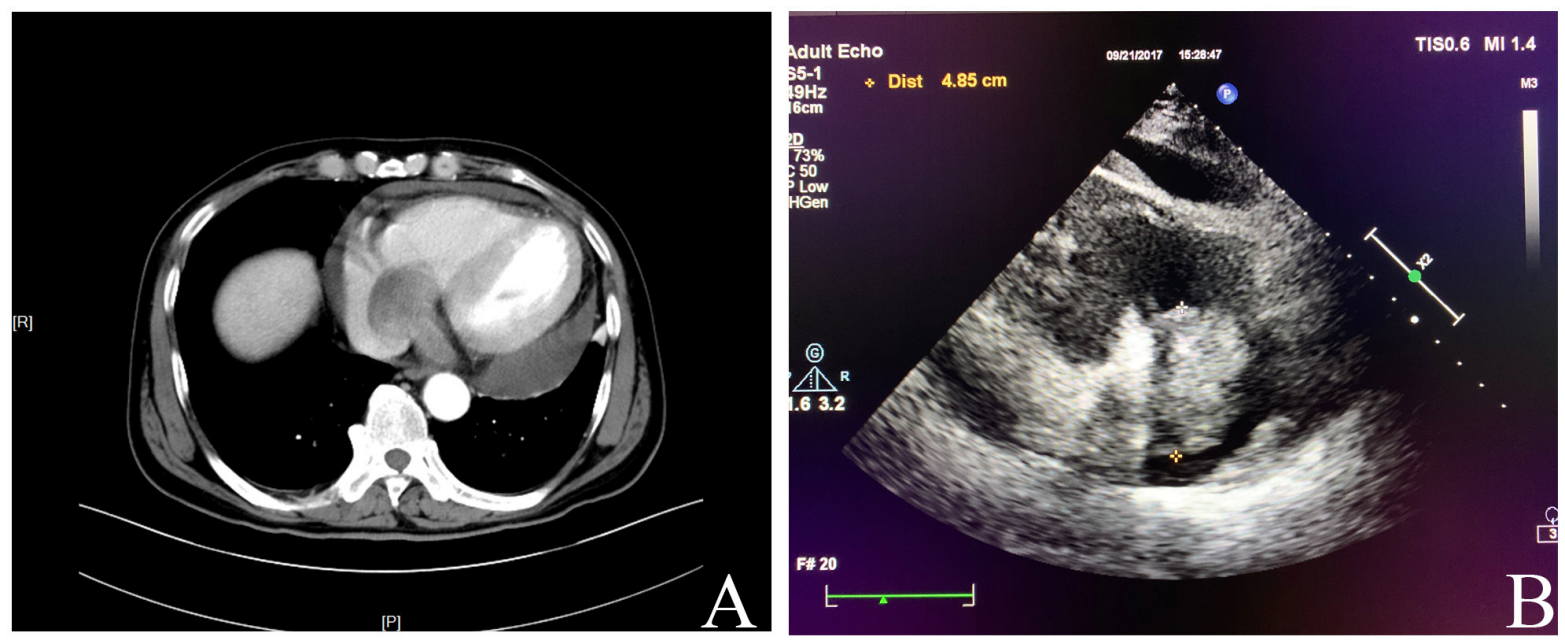
**(A)** Computed tomography showed an occupying lesion involving the right atrium and the right ventricle. **(B)** Echocardiography revealed a mass in the right atrium with moderate pericardial effusion.

A 62-year-old male was admitted to our hospital complaining of chest distress and breathing difficulty while lying down at night for over 1 month. He had a medical history of hypertension and denied any specific family history. On physical examination, no heart murmurs or vascular abnormalities were audible with the heart rate of 66 beats per minute. Pulse deficit was not found as well. The abnormal laboratory findings included a modestly raised level of CA125 (36.10 U/ml) and a remarkably elevated ferritin (1,650 ng/ml). The brain natriuretic peptide level was within the normal range. Unfortunately, details on troponin and myohemoglobin levels were unavailable. An electrocardiogram indicated left ventricular hypertrophy and complete right bundle branch block. A computed tomography (CT) scan showed an occupying lesion involving the right atrium and the right ventricle (Figure 1A) without any abdominal abnormalities. The echocardiography revealed a mass in the right atrium with moderate pericardial effusion (Figure 1B). The patient then underwent a surgical resection. The operation findings suggested that the aorta and the pulmonary artery were normally arranged, with the diameter of the aorta being about 2.8 cm and the pulmonary artery at 3.0 cm. A mass of the size 3 × 3 × 2 cm was located on the right ventricular diaphragmatic surface, infiltrating the ventricular myocardium without a clear demarcation, which made excision impossible. The mass also involved the right atrium *via* the coronary sinus, forming another lesion of 4 × 3 × 3 cm that was later removed.

**Figure 2:**
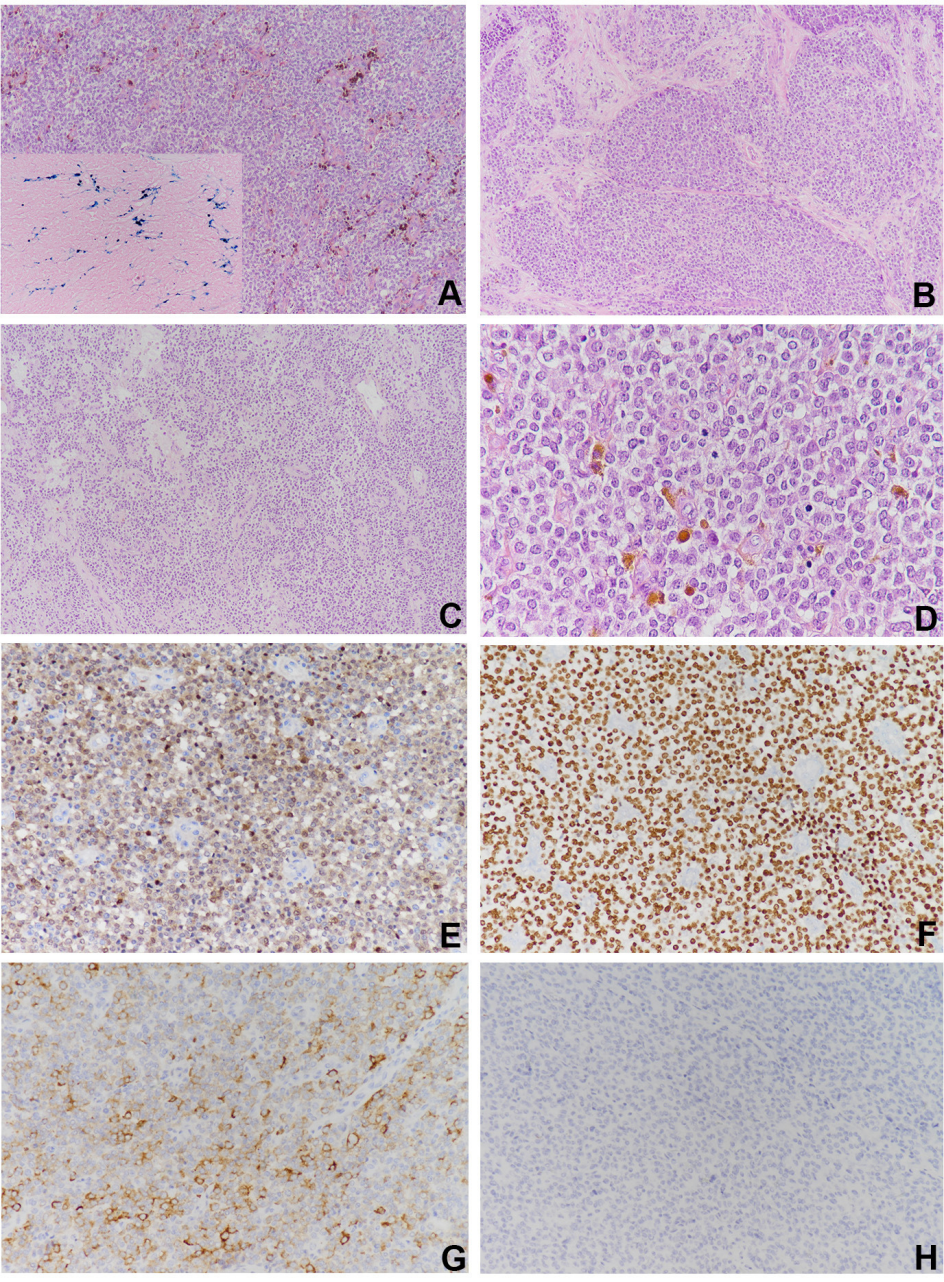
Histological findings and immunohistochemical staining of the cardiac lesion. **(A)** The neoplastic cells proliferated diffusely in the pattern of nests and sheets with scattered deposition of pigment (×100). **(B)** Fibrous separation was notable (×100). **(C)** Focal areas with remarkable cellular dyscohesion imparted a vague pseudopapillary pattern (×100). **(D)** The tumor cells were small to medium in size with fine chromatin and predominantly pale eosinophilic cytoplasm. The nuclei were typically round to oval with somewhat irregular contours and contained small nucleoli. Cytoplasmic clearing was also observed. The mitotic figures were easily found (×400). The tumor cells were strongly and diffusely positive for **(E)** S100 and **(F)** SOX10 (×100), **(G)** moderately positive for Syn, and **(H)** negative for HMB45.

**Figure 3:**
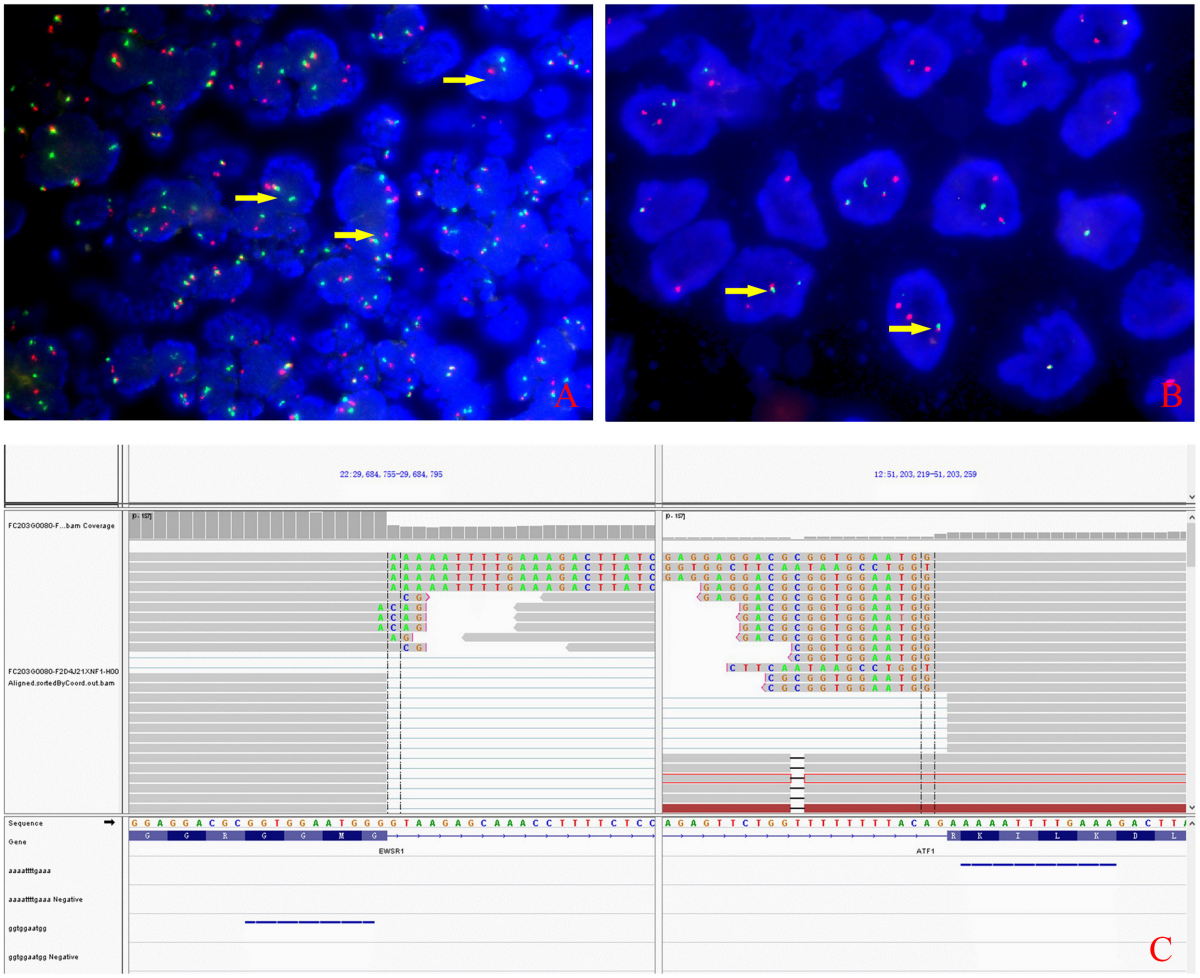
Molecular characteristics of the lesion. **(A)** The rearrangement of *EWSR1* (arrows) was found in about 50% of tumor cells by fluorescence *in situ* hybridization using *EWSR1* break-apart probe. **(B)** *EWSR1-AFTF1* dichromatic fusion probe also proved the tumor carrying the *EWSR1* rearrangement (arrows). **(C)** Whole-transcriptome sequencing analysis confirmed that the fusion involved exon 8 of EWSR1 and exon 4 of *ATF1*.

Grossly, the resected atrial lesion was soft and gray–red. Microscopically, neoplastic cells proliferated diffusely in the pattern of nests and sheets with fibrous separation (Figures 2A,B). Focal areas with remarkable cellular dyscohesion imparted a vague pseudopapillary pattern (Figure 2C). These tumor cells were small to medium in size with fine chromatin and predominantly pale eosinophilic cytoplasm. The nuclei were typically round to oval with somewhat irregular contours and contained small nucleoli (Figure 2D). The mitotic figures were easily found. Cytoplasmic clearing was also observed in this case, while osteoclast-like multinucleated giant cells were not identified (Figure 2D). In addition, deposition of hemosiderin was found as proved by iron staining (Figure 2A). Immunohistochemically, the neoplastic cells were strongly and diffusely positive for S100 (Figure 2E) and SOX-10 (Figure 2F) and moderately positive for Syn (Figure 2G), while they were negative for HMB45 (Figure 2H), A103, and CD99. The expression of INI1 was also retained, and Ki-67 stained about 40%. Fluorescence *in situ* hybridization (FISH) preformed on paraffin-embedded mass tissues identified *EWSR1*–*ATF1* rearrangement by *EWSR1* break-apart probe (Figure 3A) and *EWSR1*–*ATF1* dichromatic fusion probe (Figure 3B), respectively. Furthermore, the whole-transcriptome sequencing analysis confirmed that this fusion involved exon 8 of *EWSR1* and exon 4 of *ATF1* (Figure 3C).

**Figure 4:**
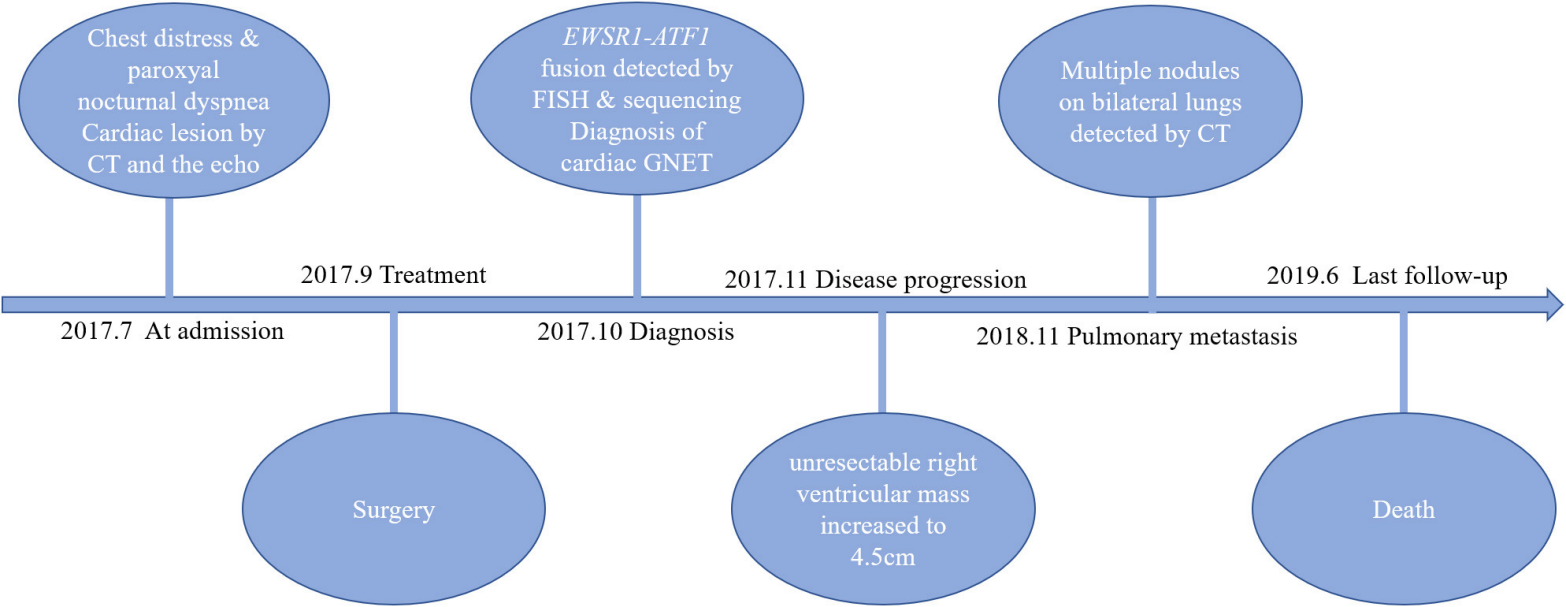
Timeline of this study.

The second full-body CT scan carried out after the surgery displayed no abnormality other than the previous unresected right ventricular mass. The patient also underwent GI endoscopy as advised, which demonstrated no lesions. Finally, a diagnosis of primary cardiac GNET was established based on clinical manifestations, morphology, immunophenotype, and genetic findings. The patient refused further chemotherapy or radiotherapy, and the unresectable right ventricular mass increased to 4.5 cm in diameter, suggesting disease progression just 1 month after the surgery. Then, he had pulmonary metastasis 8 months later and soon died of the disease in less than a year. The overall survival of the patient was 20 months. The timeline is shown in Figure 4.

## Discussion

Cardiac malignancies are uncommon, and the most frequent primary cardiac sarcoma were intimal sarcomas (IS) and angiosarcomas (5). To our knowledge, GNET, as a rare soft tissue sarcoma, has never been reported in the heart before.

It is well-known that GNET is a unique clinicopathological entity harboring *EWSR1*–*ATF1/CREB1* fusions, and it can arise at any location in the GI tract, with the small intestine (particularly ileum) being the most common (4, 6). According to published literatures, only a minority of cases originated from the upper aerodigestive tract (e.g., tongue, parapharyngeal space, and bronchia) (1, 4, 7–9), which we regard as location extension in a broad sense.

As illustrated in the present case, the neoplastic cells displayed characteristic growth patterns and a strong expression of S100 and SOX-10, similar to cases with GNET in the GI tract (10–12). The absence of HMB-45 and A103 immunostaining activities suggested a lack of melanocytic differentiation, ruling out the possibility of CCS of soft tissue and melanoma. In addition, the fact that the pigment observed microscopically was proved to be hemosiderin rather than melanin also helped to exclude melanomas. The rearrangement of *EWSR1* as confirmed by FISH and whole-transcriptome sequencing further distinguished GNET from IS and malignant peripheral nerve sheath tumor (MPNST), as IS often demonstrates *MDM2* amplification and a negative expression of S100 or SOX10 (5), and MPNST seldom bears *EWSR1* variants. Ewing sarcoma/primary neuroectodermal tumor (ES/PNET) should also be considered for differentiation due to a similar expression of Syn and CD56 and morphology to GNET. However, ES/PNET often expresses CD99 protein, which was absent in this case, and frequently harbors *EWSR1-Fli1* gene fusion (13) instead of *EWSR1*–*ATF1* as detected in this case. After a full consideration of the aforementioned differential diagnoses, a diagnosis of GNET was eventually made. Moreover, both the preoperative and the postoperative full CT scan detected merely cardiac lesions, and the GI endoscopic evaluation showed no abnormalities, supporting a further diagnosis of primary cardiac GNET. We propose that this is the first GNET case reported to be located at the heart.

GNET is highly malignant and prone to develop local recurrence and metastasis (3, 4, 6). At the present, surgical resection is the mainstay of treatment for GNET, and a small number of patients have been reported to show a partial response to apatinib and anlotinib (6). In this case, the disease rapidly progressed, with the unresectable right ventricular mass enlarging obviously just 1 month after the surgery and metastasizing 8 months later, suggesting its aggressiveness and poor prognosis. As we know, metastases most frequently go to the regional lymph nodes and the liver in GNET; in this case, however, the patient had pulmonary metastasis and soon died of the disease. The overall survival of the patient was 20 months. For the time being, the optimal clinical management of GNET has not been fully established, which is attributed to the rarity of reported cases. As the patient in this case refused any further treatment after surgery, we were thus unable to offer a sound advice on clinical strategy in cardiac GNET. More clinical and experimental studies are warranted to better manage this disease in the future.

## Conclusion

In summary, we reported the first case of cardiac GNET indicating that the location of GNET should not be confined to GI tract as initially defined. It shed new light on locations primarily involved in GNET. Due to the lack of specific effective treatment and the occurrence of early metastasis, cardiac GNET conferred a poor prognosis, which clinicians should keep alert.

## Data Availability Statement

The data that support the findings of this study have been deposited into CNGB Sequence Archive of China National GeneBank DataBase with accession number CNP0002112. They were also deposited in a publicly accessible repository https://figshare.com/articles/dataset/KA49/15147315.

## Ethics Statement

The studies involving human participants were reviewed and approved by Institutional Review Board of the Affiliated Drum Tower Hospital, Nanjing University Medical School (Nanjing). The patients/participants provided their written informed consent to participate in this study. Written informed consent was obtained from the individual(s) for the publication of any potentially identifiable images or data included in this article.

## Author Contributions

ZL, XP, and LH analyzed and interpreted the data and contributed to the writing of the manuscript. YF and LL collected all the clinical and pathological data. YX and WG collected and edited the images. XF revised the final manuscript. All the authors contributed to the article and approved the submitted version.

## Conflict of Interest

The authors declare that the research was conducted in the absence of any commercial or financial relationships that could be construed as a potential conflict of interest.

## Publisher's Note

All claims expressed in this article are solely those of the authors and do not necessarily represent those of their affiliated organizations, or those of the publisher, the editors and the reviewers. Any product that may be evaluated in this article, or claim that may be made by its manufacturer, is not guaranteed or endorsed by the publisher.

## Abbreviations

GNET: malignant gastrointestinal neuroectodermal tumor
CCS: clear cell sarcoma
GI: gastrointestinal
CCSLTGT: clear cell sarcoma-like tumor of the gastrointestinal tract
CT: computed tomography
FISH: fluorescence *in situ* hybridization
IS: intimal sarcomas
MPNST: malignant peripheral nerve sheath tumor
ES/PNET: Ewing sarcoma/primary neuroectodermal tumor.
